# Importance of Fatty Acid Compositions in Patients with Peripheral Arterial Disease

**DOI:** 10.1371/journal.pone.0107003

**Published:** 2014-09-05

**Authors:** Milan Gautam, Atsushi Izawa, Yuji Shiba, Hirohiko Motoki, Takahiro Takeuchi, Ayako Okada, Takeshi Tomita, Yusuke Miyashita, Jun Koyama, Uichi Ikeda

**Affiliations:** Department of Cardiovascular Medicine, Shinshu University School of Medicine, Matsumoto, Nagano, Japan; University of Florida, United States of America

## Abstract

**Objective:**

Importance of fatty acid components and imbalances has emerged in coronary heart disease. In this study, we analyzed fatty acids and ankle-brachial index (ABI) in a Japanese cohort.

**Methods:**

Peripheral arterial disease (PAD) was diagnosed in 101 patients by ABI ≤0.90 and/or by angiography. Traditional cardiovascular risk factors and components of serum fatty acids were examined in all patients (mean age 73.2±0.9 years; 81 males), and compared with those in 373 age- and sex-matched control subjects with no evidence of PAD.

**Results:**

The presence of PAD (mean ABI: 0.71±0.02) was independently associated with low levels of gamma-linolenic acid (GLA) (OR: 0.90; 95% CI: 0.85–0.96; *P* = 0.002), eicosapentaenoic acid∶arachidonic acid (EPA∶AA) ratio (OR: 0.38; 95% CI: 0.17–0.86; *P* = 0.021), and estimated glomerular filtration rate (OR: 0.97; 95% CI: 0.96–0.98; *P*<0.0001), and with a high hemoglobin A1c level (OR: 1.34; 95% CI: 1.06–1.69; *P* = 0.013). Individuals with lower levels of GLA (≤7.95 µg/mL) and a lower EPA∶AA ratio (≤0.55) had the lowest ABI (0.96±0.02, *N* = 90), while the highest ABI (1.12±0.01, *N* = 78) was observed in individuals with higher values of both GLA and EPA∶AA ratio (*P*<0.0001).

**Conclusion:**

A low level of GLA and a low EPA∶AA ratio are independently associated with the presence of PAD. Specific fatty acid abnormalities and imbalances could lead to new strategies for risk stratification and prevention in PAD patients.

## Introduction

Peripheral arterial disease (PAD) is a manifestation of advanced systemic atherosclerosis, and is associated with high mortality and morbidity from cardiovascular disease (CVD) [1], [2]. The prevalence of PAD increases with age in both sexes, and affects 4.3% of individuals aged ≥40 years and 14.5% of those aged ≥70 years in the United States [3]. Although the prevalence of PAD in Japanese aged ≥40 years is lower, ranging from 1.5% to 2.7% [4], [5], [6], the number of Japanese PAD patients is anticipated to increase in the near future.

Traditional risk factors, such as diabetes mellitus (DM), hypertension, current smoking, and dyslipidemia, are strongly associated with the risk of PAD [3], [7]. In addition, other biomarkers, including homocysteine, C-reactive protein (CRP), lipoprotein(a), fibrinogen, and apolipoproteins (apo) A-I and B-100 have been examined for their association with PAD [7], [8], [9]. These biomarkers are not consistently and independently associated with the presence of PAD [8], [9], [10], [11]; therefore, it would be of great interest if new markers in addition to the traditional risk factors could contribute substantially to the diagnosis and the management of PAD.

A large scale cohort study spanning 30,829 person-years demonstrated that higher circulating components of omega-3 polyunsaturated fatty acid (PUFA) or the total omega-3 level were associated with lower total mortality, especially deaths from coronary heart disease in healthy older adults [12]. Likewise, low levels of omega-6 PUFAs (linoleic acid [13], [14] or dihomo-gamma-linolenic acid [15]) have been linked to the risk of coronary heart disease. Moreover, a reduced ratio of eicosapentaenoic acid (EPA), an omega-3 PUFA, to arachidonic acid (AA), an omega-6 PUFA (EPA∶AA), has recently been highlighted as a risk predictor for secondary cardiovascular events [16], [17], [18], [19]. Importantly, these abnormalities and/or imbalances can be a therapeutic target to prevent future cardiovascular events; however, some controversy still exists regarding the efficacy of individual fatty acids in the prevention of adverse cardiovascular outcomes. Recently, one large-scale randomized clinical trial, as well as a systemic review and meta-analyses of randomized clinical trials, failed to show any efficacy of omega-3 PUFA supplementation in reducing cardiovascular mortality [20], [21]. Overall, the effects of omega-3 and omega-6 PUFAs in the PAD population have remained elusive.

The Framingham risk score [22] and the NIPPON DATA80 [23] chart in Japan have been used to assess the 10-year risk of cardiovascular mortality in the general population; however, factors associated with the presence of PAD have not been completely identified. In addition, current management is not sufficient to fully minimize future cardiovascular events in PAD patients. In this case-controlled study, we examined well-known cardiovascular risk factors and fatty acid composition in PAD patients, in an attempt to provide a new strategy for their risk management.

## Methods

### 1. Study design

This case-control study enrolled adults aged 40 years and older with PAD. PAD was defined if a patient had a resting ankle-brachial index (ABI) ≤0.90 in at least one limb, and/or in claudicant patients who had one or more stenoses >75% in at least one leg artery on angiography. Patients receiving oral EPA supplements and patients with chronic kidney disease (CKD) undergoing hemodialysis were excluded from the study. Laboratory analyses and ABI levels of the cases were compared with those of age- and sex-matched control subjects who had ABI levels between 0.90 and 1.40 or no evidence of PAD. The presence of comorbid diseases was determined as follows: 1) coronary heart disease was diagnosed by coronary angiography, 2) diabetes mellitus by an elevation of fasting blood glucose level (≥126 mg/dL) or use of antidiabetic agents, 3) dyslipidemia by the criteria of the Japan Atherosclerosis Society [24] or use of lipid-lowering agents, and 4) CKD by estimated glomerular filtration rate (eGFR) <60 mL/min/1.73 m^2^. Levels of eGFR were calculated using the Modified Diet in Renal Disease study equation, modified for Japanese patients with CKD: eGFR  = 194 × Serum creatinine^−1.094^×Age^−0.287^ (×0.739 if female) mL/min/1.73 m^2^ [25].

The ethics committee of Shinshu University School of Medicine approved the protocol. The study was performed in accordance with the Declaration of Helsinki and with Good Clinical Practice. Patients were managed according to the recommended guidelines of the Japanese Circulation Society.

### 2. Laboratory analyses

Levels of low-density lipoprotein (LDL) cholesterol, high-density lipoprotein (HDL) cholesterol, triglycerides, hemoglobin A1c (HbA1c), CRP, and D-dimer were measured using routine laboratory tests in a hospital laboratory. Components of fatty acid levels were estimated from serum samples of fasting venous blood in all patients and control subjects using a gas chromatography technique, as described previously, at Special Reference Laboratories, Inc., Tokyo, Japan [16].

### 3. Measurements of ABI

Systolic blood pressures in upper extremities (brachial artery) and lower extremities (dorsalis pedis and posterior tibial artery) were measured using a FORM ABI/PWV device (Omron Colin Co., Tokyo, Japan), with the subjects resting in a supine position for at least 10 minutes. The ABI is the ratio of the systolic blood pressure in the leg to that in the arms. The ABI was calculated separately for each leg and the lower measurement was used in the analyses; an ABI ≤0.90 at rest represents clinically significant PAD.

### 4. Statistical analyses

Data were analyzed anonymously by using IBM SPSS Statistics version 18 (IBM Co.) and GraphPad Prism version 5.0f (GraphPad Software, San Diego, California). Throughout the analysis, a two-tailed *P* value <0.05 was considered to be statistically significant. Differences in continuous variables were assessed by Mann–Whitney *U* tests, and Fisher's exact tests were used to examine differences in categorical variables between the cases and the controls. Univariable logistic regression analyses were performed on the significant variables in the non-parametric tests, and followed by multivariable-adjusted logistic regression analyses for significant variables in the univariable model. Odds ratios (OR) and their 95% confidence intervals (CI) were calculated, with adjustments for age, sex, body mass index, eGFR, HbA1c, gamma-linolenic acid (GLA), EPA∶AA ratio, and docosahexaenoic∶arachidonic acid (DHA∶AA) ratio. Mann–Whitney *U* tests was used to calculate the distributions of GLA and EPA∶AA ratio according to ABI levels. The areas under the receiver operating characteristic (ROC) curve were used to identify the sensitivity and specificity of cut-off points for the detection of PAD. We next calculated cut-off points at which the value of “sensitivity + specificity – 1” was maximum (Youden's Index). One way ANOVA with Tukey's multiple comparison test was used to compare ABI levels in individuals with lower or higher proportions of GLA and EPA∶AA ratio using the cutoff values calculated from the ROC curve analyses.

## Results

### 1. Characteristics of the study population

A total of 474 patients (101 cases with PAD and 373 controls without PAD) were enrolled. Among all subjects, mean age was 72.6±0.4 years, and 82% were male. The baseline characteristics are summarized in Table 1.

**Table 1:** Baseline characteristics of the study population.

Patient's characteristics	PAD (*N* = 101)	Controls (*N* = 373)	*P* value*
Age (years)	73.2±0.9	72.4±0.4	0.243
Sex (male), *N* (%)	81 (80)	306 (82)	0.665
Body mass index (kg/m^2^)	22.45±0.33	23.61±0.16	0.001
Triglycerides (mg/dL)	136.5±7.35	142.3±3.87	0.245
LDL-C (mg/dL)	95.67±3.26	98.76±1.57	0.245
HDL-C (mg/dL)	48.69±1.48	51.16±0.75	0.115
LDL∶HDL ratio	2.14±0.10	2.03±0.04	0.702
ABI	0.71±0.02	1.12±0.005	<0.0001
CRP (mg/dL)	0.44±0.09	0.28±0.04	0.008
D-dimer (µg/mL)	2.16±0.33	1.80±0.19	<0.0001
HbA1c (%)	6.45±0.12	6.02±0.07	0.002
eGFR (mL/min/1.73 m^2^)	49.96±2.37	64.57±0.95	<0.0001
Statin, *N* (%)	48 (48)	211 (57)	0.1153
**Comorbidity, ** ***N*** ** (%)**			
Coronary heart disease	61 (60)	245 (66)	0.349
Diabetes mellitus	46 (46)	123 (33)	0.025
CKD (eGFR <60 mL/min/1.73 m^2^)	59 (58)	139 (37)	0.0002
Dyslipidemia	18 (18)	93 (25)	0.146

Abbreviations: PAD, peripheral artery disease; LDL-C, low-density lipoprotein cholesterol; HDL-C high-density lipoprotein cholesterol; ABI, ankle-brachial index; CRP, c-reactive protein; HbA1c, hemoglobin A1c; eGFR, estimated glomerular filtration rate; CKD, chronic kidney disease. Data are given as mean ± standard error of mean or n (%);

*The Mann–Whitney *U* test and Fisher's exact test were used to analyze differences in continuous and categorical variables, respectively.

### 2. Conventional risk factors

There were no significant differences in the baseline serum levels of triglyceride, HDL cholesterol, LDL cholesterol, or LDL∶HDL cholesterol ratio between the cases and the controls. No significant differences were observed between the cases and the controls in the numbers of patients with coronary heart disease and dyslipidemia. There was significantly higher morbidity from diabetes (46% versus 33%, *P* = 0.025) and CKD (58% versus 37%, *P* = 0.0002) in the PAD patients than in the controls. Levels of D-dimer and CRP were also significantly elevated in PAD patients compared to controls (*P*<0.05) (Table 1).

### 3. Fatty acid components

**Figure 1:**
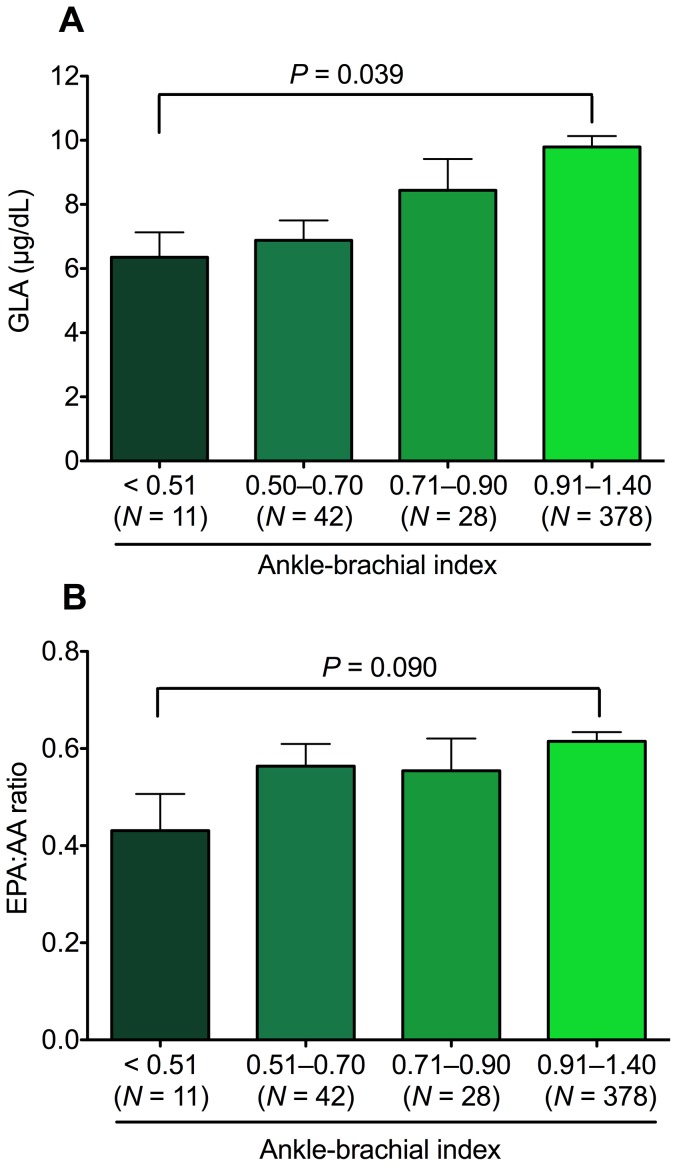
Distribution of levels of (A) gamma-linolenic acid (GLA) and (B) eicosapentaenoic acid∶arachidonic acid (EPA∶AA) ratio according to ankle-brachial index in 459 subjects.

The PAD patients had significantly lower levels of docosapentaenoic acid (12%), docosahexaenoic acid (7%), GLA (27%), EPA∶AA ratio (10%), and DHA∶AA ratio (11%) than the controls. The lower levels of EPA∶AA ratio and DHA∶AA ratio were mainly due to lower EPA and DHA concentrations in PAD group. However, no significant differences were observed between the groups in saturated fatty acids, monosaturated fatty acids, alpha-linolenic acid, linoleic acid, dihomo-gamma-linolenic acid, AA, or eicosadienoic acid (Table 2). The distributions of GLA and EPA∶AA ratio according to ABI levels were further examined (*N* = 459). The GLA level in patients with ABI<0.51 was 6.35±0.77, significantly lower than the 9.79±0.33 in patients with ABI>0.90 and ≤1.40 (*P* = 0.039; Fig. 1A). The EPA∶AA ratio in patients with ABI<0.51 was 0.43±0.07, modestly lower than the 0.61±0.01 in patients with ABI>0.90 and ≤1.40 (*P* = 0.090; Fig. 1B).

**Table 2:** Serum fatty acid composition.

Fatty Acids (µg/mL)	PAD (*N* = 101)	Controls (*N* = 373)	*P* value*
**Saturated**			
Lauric acid (12∶0)	5.1±1.45	4.45±0.40	0.644
Myristic acid (14∶0)	31.59±2.49	33.23±1.10	0.090
Palmitic acid (16∶0)	695.9±23.57	707.6±11.99	0.536
Stearic acid (18∶0)	205.3±6.04	215.4±3.19	0.111
Behenic acid (22∶0)	15.81±0.46	16.58±0.46	0.133
Lignoceric acid (24∶0)	15.30±0.45	15.87±0.64	0.588
**Monounsaturated**			
Arachidic acid (20∶0)	7.14±0.17	7.23±0.08	0.704
Palmitoleic acid (16∶1)	70.60±4.12	69.65±1.97	0.833
Eicosenoic acid (20∶1)	6.53±0.29	6.72±0.22	0.627
Olieic acid (18∶1)	627.5±26.32	616.3±12.0	0.814
Erucic acid (22∶1)	1.90±0.13	1.97±0.26	0.299
**Polyunsaturated**			
Omega-3			
Alpha-linolenic acid (18∶3)	28.71±1.64	30.11±0.86	0.284
EPA (20∶5)	76.71±4.32	89.81±2.44	0.008
DHA (22∶6)	150.2±7.11	163.7±3.01	0.012
Docosapentaenoic acid (22∶5)	21.60±0.98	23.77±0.51	0.024
Omega-6			
Linoleic acid (18∶2)	772.8±25.55	775.2±11.13	0.490
GLA (18∶3)	7.58±0.42	10.07±0.33	< 0.0001
Dihomo-gamma-linolenic acid (20∶3)	31.66±1.23	33.57±0.65	0.128
AA (20∶4)	155.6±5.04	157.1±2.55	0.652
Docosatetraenoic acid (22∶4)	4.55±0.18	4.52±0.12	0.596
Eicosadienoic acid (20∶2)	5.92±0.20	5.88±0.09	0.612
EPA∶AA ratio	0.53±0.03	0.62±0.01	0.020
DHA∶AA ratio	1.01±0.04	1.10±0.02	0.020

Abbreviations: PAD, peripheral artery disease; EPA, eicosapentaenoic acid; DHA, docosahexaenoic acid; GLA, gamma-linolenic acid; AA, arachidonic acid; EPA∶AA, eicosapentaenoic acid to arachidonic acid ratio; DHA∶AA, docosahexaenoic acid to arachidonic acid ratio. Numbers in parentheses show number of carbons and double bonds. Data are given as mean ± standard error of mean;

*Mann Whitney *U* test.

### 4. Factors associated with the presence of PAD

**Figure 2:**
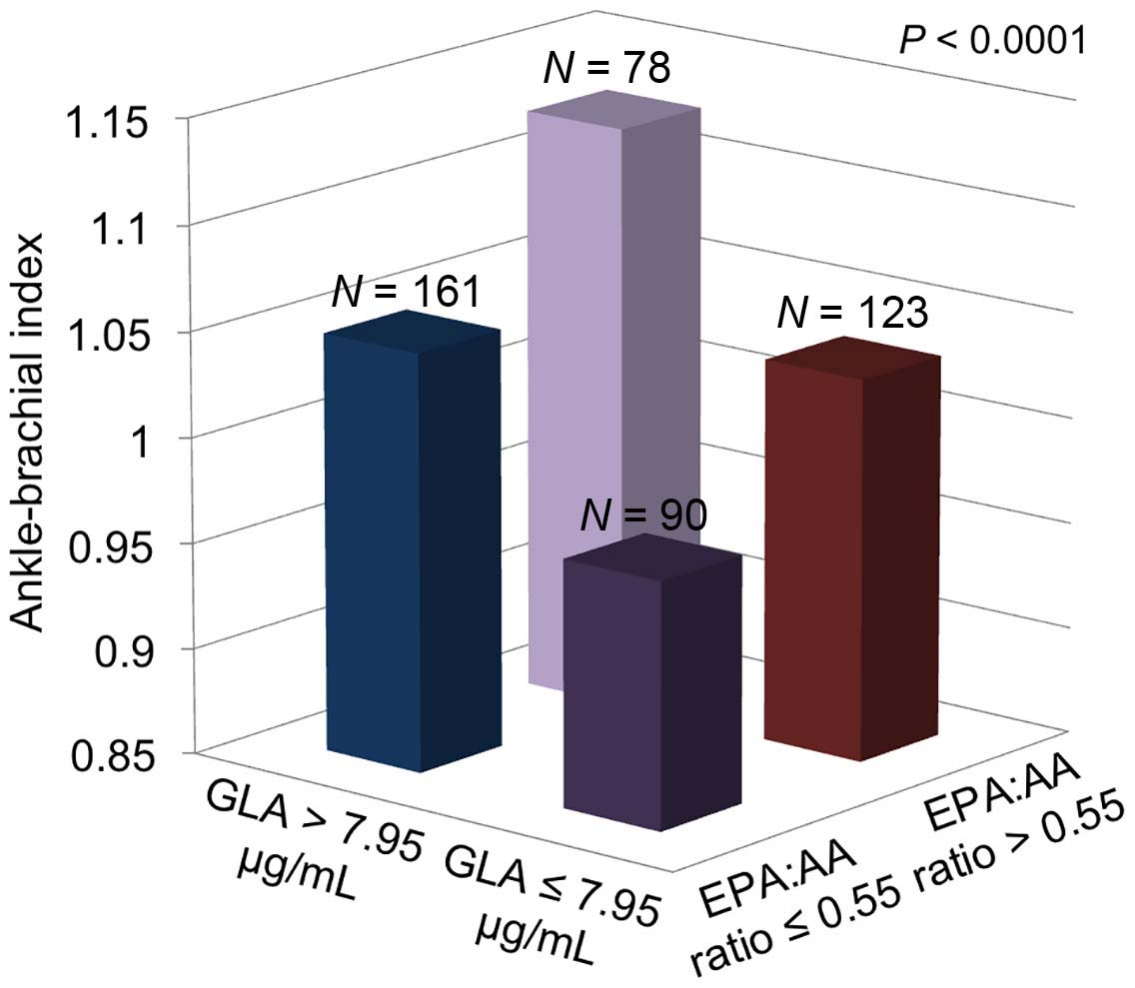
The association of serum levels of gamma-linolenic acid (GLA) and eicosapentaenoic acid∶arachidonic acid (EPA∶AA) ratio with ankle-brachial index levels. Study population was divided into 4 groups by using cut-off values at 7.95 µg/mL of GLA and at 0.55 of EPA∶AA ratio. There was an association of low levels of GLA and low EPA∶AA ratios with the presence of PAD.

Univariable logistic regression analyses showed that the presence of PAD was associated with low body mass index, reduced eGFR, elevated HbA1c and low levels of EPA, GLA, and EPA∶AA ratio (*P*<0.05). In analyses using multivariable-adjusted logistic regression, the presence of PAD was independently associated with reduced eGFR, elevated HbA1c, and low GLA levels and EPA∶AA ratio (*P*<0.05; Table 3). The areas under the ROC curve (95% CI) for eGFR, HbA1c, GLA, and EPA∶AA ratio were 0.67 (0.60–0.73, *P*<0.0001), 0.59 (0.53–0.66, *P* = 0.002), 0.63 (0.57–0.69, *P*<0.0001), and 0.58 (0.51–0.64, *P* = 0.020), respectively. Based on the ROC curve analyses, the optimal cutoff values for eGFR, HbA1c, GLA, and EPA∶AA ratio were 45.50 mL/min/1.73 m^2^ (42.0% sensitivity, 86.92% specificity), 6.05% (60.0% sensitivity, 58.45% specificity), 7.95 µg/mL (67.33% sensitivity, 58.49% specificity), and 0.55 (66.34% sensitivity, 47.18% specificity), respectively. Using the optimal cutoff values for GLA and EPA∶AA ratio, ABI levels were compared among the 4 subgroups (*N* = 90, GLA ≤7.95 µg/mL and EPA∶AA ratio ≤0.55; *N* = 161, GLA >7.95 µg/mL and EPA∶AA ratio ≤0.55; *N* = 123, GLA ≤7.95 µg/mL and EPA∶AA ratio >0.55; *N* = 78, GLA >7.95 µg/mL and EPA∶AA ratio >0.55; Fig. 2). There was a significant difference in ABI levels among the 4 subgroups (*P*<0.0001). Furthermore, the mean level of ABI (0.96±0.02) was lowest in individuals with a lower GLA (≤7.95 µg/mL) and a lower EPA∶AA ratio (≤0.55), as compared with the highest ABI levels (1.12±0.01) observed in individuals with higher values of both GLA and EPA∶AA ratio (*P*<0.001).

**Table 3:** Logistic regression analyses of 101 cases of PAD and 373 control subjects.

Variables	Univariable analysis		Multivariable analyses*	
	OR (95% CI)	*P* value	OR (95% CI)	*P* value
Age	1.01 (0.98–1.04)	0.386	0.99 (0.95–1.02)	0.532
Sex (male)	1.12 (0.64–1.96)	0.672	1.06 (0.56–1.99)	0.854
Body mass index	0.89 (0.83–0.96)	0.002	0.92 (0.85–1.002)	0.058
eGFR	0.96 (0.95–0.97)	<0.0001	0.97 (0.96–0.98)	<0.0001
HbA1c	1.28 (1.06–1.55)	0.009	1.34 (1.06–1.69)	0.013
CRP	1.19 (0.95–1.48)	0.199		
D-dimer	1.02 (0.96–1.08)	0.397		
EPA	0.99 (0.98–0.99)	0.013		
DHA	0.99 (0.99–1)	0.051		
Docosapentaenoic acid	0.97 (0.95–1)	0.054		
GLA	0.90 (0.85–0.95)	<0.0001	0.90 (0.85–0.96)	0.002
EPA∶AA ratio	0.45 (0.22–0.91)	0.026	0.38 (0.17–0.86)	0.021
DHA∶AA ratio	0.57 (0.33–1.005)	0.052	0.59 (0.31–1.11)	0.104

Abbreviations: PAD, peripheral artery disease; eGFR, estimated glomerular filtration rate; HbA1c, hemoglobin A1c; CRP, c-reactive protein; EPA, eicosapentaenoic acid; DHA, docosahexaenoic acid; GLA, gamma-linolenic acid; EPA∶AA, eicosapentaenoic acid to arachidonic acid ratio; DHA∶AA, docosahexaenoic acid to arachidonic acid ratio; OR, odds ratio; CI, confidence interval.

*Adjusted for age, sex, body mass index, eGFR, HbA1c, and GLA.

Our results suggested that combined low GLA levels and low EPA∶AA ratios were correlated with a potentially more advanced status of PAD. There were 90 enrolled patients with combined low GLA levels (≤7.95 µg/mL) and low EPA∶AA ratios (≤0.55), and these patients had high morbidity of coronary heart disease (69%), chronic kidney disease (59%), diabetes mellitus (41%), and dyslipidemia (24%). Around 54% of these patients received statin treatment.

## Discussion

This study showed that a low level of GLA and a low EPA∶AA ratio were independently associated with the presence of PAD. Moreover, individuals with both a low GLA level and a low EPA∶AA ratio had the lowest ABI levels, which suggest an advanced status of PAD. The areas under the ROC curves for CKD, DM, GLA, and EPA∶AA ratio were comparable, suggesting that these values had equally important associations with the presence of PAD. In contrast, neither the other components of fatty acids nor the DHA∶AA ratio had a significant association with PAD.

Background diseases in Japanese PAD patients have shown a high prevalence of DM (41.2%) [26] and CKD (60.7%) [27]. Similarly, high morbidities of DM and CKD were observed in PAD patients in this study. The average body mass index of our PAD patients was 5% lower than that of controls. This suggested the obesity paradox, which has been explained in part by malnutrition and/or systemic inflammation due to the severity of PAD and, more importantly, is associated with the high mortality of PAD patients [28], [29]. In fact, PAD patients in this study had significantly high CRP levels.

The following differences between EPA and AA have been demonstrated: EPA-derived eicosanoids decrease plasma triglyceride levels, improve endothelial function and arterial stiffness, inhibit platelet aggregation, attenuate inflammation, and stabilize atheromatous plaque [30], [31], [32], [33], [34], whereas AA derived eicosanoids are pro-inflammatory, pre-thrombotic, and vasoconstrictive [31], [32]. Importantly, the benefits of fish intake or dietary supplements of omega-3 PUFAs [16], [35], [36], [37] have emerged in large-scale randomized clinical trials with EPA that reduced coronary events by 19% in Japanese hypercholesterolemic patients [37]. Additive administration of EPA to increase the EPA∶AA ratio has reduced the incidence of secondary major adverse cardiovascular events in patients with either PAD [38] or established coronary artery disease [18].

Several findings that indicate favorable effects of GLA (omega-6 PUFA) on cardiovascular diseases have been reported. GLA supplementation was shown to have anti-inflammatory or immunomodulatory roles in patients with multiple sclerosis [39] or acne vulgaris [40], and also to promote vasodilation [41], reduce blood pressure [42], or to have an inhibitory effect on smooth muscle cell proliferation associated with the progression of atherosclerosis [43]. Combined treatment with GLA and EPA for 2 years in 120 patients with intermittent claudication showed a significant reduction in systolic blood pressure (*P*≤0.05), and modestly reduced non-fatal coronary events (10% vs. 15%, *P*>0.05) [44]. These reports could support the results of this study, which suggest a potential role of GLA in inflammation mediating the development of peripheral atherosclerosis.

Current clinical guidelines recommend consumption of omega-3 PUFAs for secondary prevention [45], [46]. Japan has one of the highest mean dietary omega-3 PUFAs intake in the world [47]. More cardiovascular benefits of omega-3 PUFAs would be expected in countries with low omega-3 PUFAs consumption. In addition to the benefits of omega-3 PUFAs, an American Heart Association Science Advisory has recommended the consumption of omega-6 PUFAs of at least 5% to 10% of energy to reduce the risk of coronary artery disease [48]. In fact, a recent case control study demonstrated that patients with a recent myocardial infarction had lower total PUFA levels of both omega-3 and omega-6, as compared with those in control subjects [49]. In our study, however, only GLA levels, but no other PUFA components of omega-6 or the total level, were significantly associated with PAD. Further studies are required to clarify whether a lower GLA level has an exclusive role in the pathophysiology of PAD.

Lipid-lowering therapies with strong statins have been shown to modify the concentrations of omega-3 and omega-6 PUFAs [50], [51]; however, we observed no significant difference in the proportion of individuals receiving statin therapy or in lipid profiles between PAD patients and control subjects (Table 1). In addition, neither GLA levels nor the EPA∶AA ratio were significantly correlated with lipid profiles in our study.

There are some limitations to our study. First, the sample size was relatively small and the study was performed at a single facility using hospital controls to minimize confounding variables. Second, the attending physician based the diagnosis of PAD on the diagnostic criteria, while their clinical manifestations and disease severity were not reported. Third, this study was not designed to calculate the amount of fish consumption and/or omega-3 intake in the study population. Fourth, the mechanisms of action of fatty acid components in PAD and therapeutic strategies utilizing specific components were beyond the scope of our study. Further research is encouraged to establish risk management and treatment strategies for patients with PAD.

In conclusion, we found that low levels of GLA and a low EPA∶AA ratio were significantly associated with the presence and advanced status of PAD in Japanese. These findings imply that, in addition to the well-known cardiovascular risk factors, abnormalities and imbalance of fatty acids play an important role in the pathophysiology of peripheral atherosclerosis, and may open new strategies for risk stratification and prevention for PAD patients.
